# Survival rates and predictors of survival among colorectal cancer patients in a Malaysian tertiary hospital

**DOI:** 10.1186/s12885-017-3336-z

**Published:** 2017-05-18

**Authors:** Bello Arkilla Magaji, Foong Ming Moy, April Camilla Roslani, Chee Wei Law

**Affiliations:** 1 Julius Centre University of Malaya, Department of Social & Preventive Medicine, Faculty of Medicine, University of Malaya, 50603 Kuala Lumpur, Malaysia; 2 Department of Surgery, Faculty of Medicine, University of Malaya, 50603 Kuala Lumpur, Malaysia; 3 University of Malaya Cancer Research Institute (UMCRI), Kuala Lumpur, Malaysia; 4 Department of Community Health, College of Health Sciences, Usmanu Danfodiyo University, PMB, Sokoto, 2346 Nigeria

**Keywords:** Colorectal cancer, Survival rates, Ethnic disparities, Malaysia

## Abstract

**Background:**

Colorectal cancer is the third most commonly diagnosed malignancy and the fourth leading cause of cancer-related death globally. It is the second most common cancer among both males and females in Malaysia. The economic burden of colorectal cancer is likely to increase over time owing to its current trend and aging population. Cancer survival analysis is an essential indicator for early detection and improvement in cancer treatment. However, there was a scarcity of studies concerning survival of colorectal cancer patients as well as its predictors. Therefore, we aimed to determine the 1-, 3- and 5-year survival rates, compare survival rates among ethnic groups and determine the predictors of survival among colorectal cancer patients.

**Methods:**

This was an ambidirectional cohort study conducted at the University Malaya Medical Centre (UMMC) in Kuala Lumpur, Malaysia. All Malaysian citizens or permanent residents with histologically confirmed diagnosis of colorectal cancer seen at UMMC from 1 January 2001 to 31 December 2010 were included in the study. Demographic and clinical characteristics were extracted from the medical records. Patients were followed-up until death or censored at the end of the study (31st December 2010). Censored patients’ vital status (whether alive or dead) were cross checked with the National Registration Department. Survival analyses at 1-, 3- and 5-year intervals were performed using the Kaplan-Meier method. Log-rank test was used to compare the survival rates, while Cox proportional hazard regression analysis was carried out to determine the predictors of 5-year colorectal cancer survival.

**Results:**

Among 1212 patients, the median survival for colorectal, colon and rectal cancers were 42.0, 42.0 and 41.0 months respectively; while the 1-, 3-, and 5-year relative survival rates ranged from 73.8 to 76.0%, 52.1 to 53.7% and 40.4 to 45.4% respectively. The Chinese patients had the lowest 5-year survival compared to Malay and Indian patients. Based on the 814 patients with data on their Duke’s staging, independent predictors of poor colorectal cancer (5-year) survival were male sex (Hazard Ratio [HR]: 1.41; 95% CI: 1.12, 1.76), Chinese ethnicity (HR: 1.41; 95% CI: 1.07,1.85), elevated (≥ 5.1 ng/ml) pre-operative carcino-embryonic antigen (CEA) level (HR: 2.13; 95% CI: 1.60, 2.83), Duke’s stage C (HR: 1.68; 95% CI: 1.28, 2.21), Duke’s stage D (HR: 4.61; 95% CI: 3.39, 6.28) and emergency surgery (HR: 1.52; 95% CI: 1.07, 2.15).

**Conclusions:**

The survival rates of colorectal cancer among our patients were comparable with those of some Asian countries but lower than those found in more developed countries. Males and patients from the Chinese ethnic group had lower survival rates compared to their counterparts. More advanced staging and late presentation were important predictors of colorectal cancer survival. Health education programs targeting high risk groups and emphasizing the importance of screening and early diagnosis, as well as the recognition of symptoms and risk factors should be implemented. A nationwide colorectal cancer screening program should be designed and implemented to increase early detection and improve survival outcomes.

## Background

Colorectal cancer is the third most commonly diagnosed malignancy and the fourth leading cause of cancer-related death in the world. Its burden is expected to increase to more than 2.2 million new cases and 1.1 million cancer deaths by 2030 [1]. Rapid increases in both colorectal cancer incidence and mortality are now being observed in the Asian countries.

Malaysia is an upper middle income country with a multi-ethnic population of 31.7 million. The major ethnic groups are Malays (68.6%), Chinese (23.4%) and Indians (7%) [2]. In Malaysia, colorectal cancer is the second most common cancer in both males and females [3]. According to the National Cancer Patient Registry - Colorectal Cancer data from 2008 to 2013, the overall incidence and mortality rates of colorectal cancers among the Malaysian population were 21.32 and 9.79 per 100,000 respectively [4]. Those of Chinese ethnicity had the highest incidence of colorectal cancer (27.35 per 100,000), followed by the Malays (18.95 per 100,000), and Indians (17.55 per 10,000) [4]. The economic burden of colorectal cancer is substantial and is likely to increase over time owing to the current trend and aging population.

Mortality due to colorectal cancer can be effectively reduced with early diagnosis and treatment. However, previous studies showed that colorectal cancer patients in Malaysia usually presented late compared to developed countries [5, 6]. This could be due to the lack of a national screening program, poor appreciation of its common symptoms, risk factors and available measures for its early detection [6–8].

There was also a scarcity of studies on the treatment outcomes as well as the overall survival of colorectal cancer patients in our country [6]. Only three local studies on colorectal cancer survival outcomes with small sample sizes [9–11] were published. Cancer survival analysis is an essential indicator for early detection and improvement in cancer treatment. Therefore, we aimed to (1) determine the 1-, 3- and 5-year relative survival rates of colorectal cancer patients; (2) compare the survival rates of colorectal cancer patients among the three major ethnic groups; and (3) evaluate the roles of selected demographic, clinical and treatment factors in the prediction of survival for colorectal cancer patients treated at the University Malaya Medical Centre (UMMC) from January 2001 to December 2010.

## Methods

This was an ambidirectional cohort study, which comprised of retrospective and prospective components. All Malaysian citizens or permanent residents with histologically confirmed diagnosis of colorectal cancer seen at UMMC from 1 January 2001 to 31 December 2010 were included. The patients’ unique National Registration Identity Card (NRIC) numbers and their hospital registration numbers were used to identify and link their medical records. Data were retrieved from the patients’ medical records retrospectively from January 2001 to December 2008, and prospectively from January 2009 to December 2010.

Data extracted from the medical records included demographic characteristics such as age, sex and ethnicity (Malays, Chinese, Indians and Others) and clinical characteristics i.e.: comorbidities (such as hypertension and diabetes mellitus), anatomic site of the tumour, Duke’s stages (A to D), tumour grades and pre-operative serum carcino-embryonic antigen (CEA) levels. Treatment modalities such as surgery, chemotherapy and radiotherapy or best supportive treatment were also collected. The date of surgery or commencement of treatment in UMMC was considered as the zero date. Patients were followed-up until death or censored at the end of the study (31st December 2010). Censored patients’ NRIC numbers were used to cross check with the data of the National Registration Department (NRD) for their vital status (whether still alive or dead).

Data were analysed using SPSS, version 21.0 for Windows, (SPSS Inc., and Chicago, Illinois, USA). The level of significance was pre-set at 0.05. Quantitative variables were screened for normality. Normally distributed variables were summarized using means and standard deviations (SD), while skewed data were reported as median and inter-quartile range. Categorical variables were presented using proportions and percentages.

Survival analyses at 1-, 3- and 5-year intervals were performed using the Kaplan-Meier method. The log-rank test was used to compare the survival rates. The Cox proportional hazard regression analysis was carried out to determine the hazard ratios of predictors on survival. Variables with *p* < 0.25 in the univariate analyses were entered into the multivariate model. An automatic stepwise backward elimination method was performed. Confidence intervals of 95% were reported where appropriate.

## Results

### Patients’ characteristics, follow-up and relative survival rates

A total of 1212 eligible patients were included in the analysis. Details of the demographics, clinical and treatment characteristics of the patients are reported in Table 1. Slightly more than half of the patients were males and 67% were of Chinese ethnicity. The majority were aged more than 50 years with a mean (SD) age of 61 (13) years. There was an equal proportion of patients with colon or rectal cancers. Almost half of the patients had non-elevated pre-operative CEA levels, and two thirds had low-grade tumours. About one third of the patients’ tumours were diagnosed at Duke’s stage C, while only 5% were in Dukes’ stage A. Surgery was the most common treatment modality (82.3%), while 8.3% were on chemo- and/or radiotherapy alone; and 9.4% on best supportive care. A total of 13.4% of the surgeries were performed as emergencies. More details of these patients are published elsewhere [12].

**Table 1:** Demographic and clinical characteristics of patients (*n* = 1212) in UMMC

Characteristics		n	%
Sex	Male	668	55.1
	Female	544	44.9
Ethnic groups	Chinese	808	66.7
	Malays	225	18.6
	Indians	157	13.0
	Others	22	1.7
Age groups (years)	≤ 39	83	6.8
	40–49	121	10.0
	50–59	291	24.0
	60–69	410	34.0
	≥ 70	307	25.3
Co-morbidities	Hypertension	318	26.2
	Diabetes	196	16.2
Site	Colon	596	49.2
	Rectum	552	45.5
	Unknown	64	5.3
Pre-operative carcino-embryonic antigen (CEA) level	Not elevated (≤ 5.0 ng/ml)	543	44.8
	Elevated (> 5.0 ng/ml)	256	21.1
	Unknown	413	34.1
Tumour Grades	Low	802	66.2
	High	60	5.0
	Unknown	350	28.8
Duke’s Stages	A&B	297	24.5
	C	366	30.2
	D	151	12.5
	Unknown	398	32.8
Treatment modalities	Surgery alone	488	40.3
	Surgery plus Chemo- or Radiotherapy	393	32.4
	Surgery plus Chemo- and Radiotherapy	116	9.6
	Chemo- and Radiotherapy alone	101	8.3
	Best supportive care	114	9.4
Urgency of surgery^a^	Elective	481	48.2
(*n* = 997)	Emergency	134	13.4
	Unknown	382	38.2

^a^ *n = 215 patients on chemo- and radiotherapy alone or on best supportive care*

### Relative and median survival rates

The median follow-up was 28 months (inter quartile range 11, 59 months). The median survival for colorectal cancer was 42.00 (95% CI: 35.42–48.58) months and the 5-year survival rate was 43% (Table 2). There was no significant difference in median survival among patients with colon (42 months) or rectal (41 months) cancers. However, patients with colon cancer had higher 1-, 3- and 5-year survival rates compared to rectal cancer patients.

**Table 2:** One, three and five-year relative survival (%) and median survival (months) for colorectal cancer patients

Cancer location	Relative survival rates (%)	Median survival (months)
	(95% CI)	(95% CI)
Colorectal cancer (*n* = 1212)		42.00 (35.42–48.58)
1 year	74.92 (72.38–77.26)	
3 years	53.13 (50.20–55.97)	
5 years	42.85 (39.81, 45.84)	
Colon cancer (*n* = 596)^a^		42.00 (31.11–52.89)
1 year	77.52 (73.95–80.66)	
3 years	55.04 (50.81–59.07)	
5 years	45.00 (40.59–49.31)	
Rectal cancer (*n* = 552)^a^		41.00 (32.34–49.66)
1 year	74.09 (70.23–77.54)	
3 years	52.47 (48.13–56.62)	
5 years	41.26 (36.82–45.64)	

^a^ *Total did not add up to 1212 as there were 64 unknown cases*

**Fig. 1:**
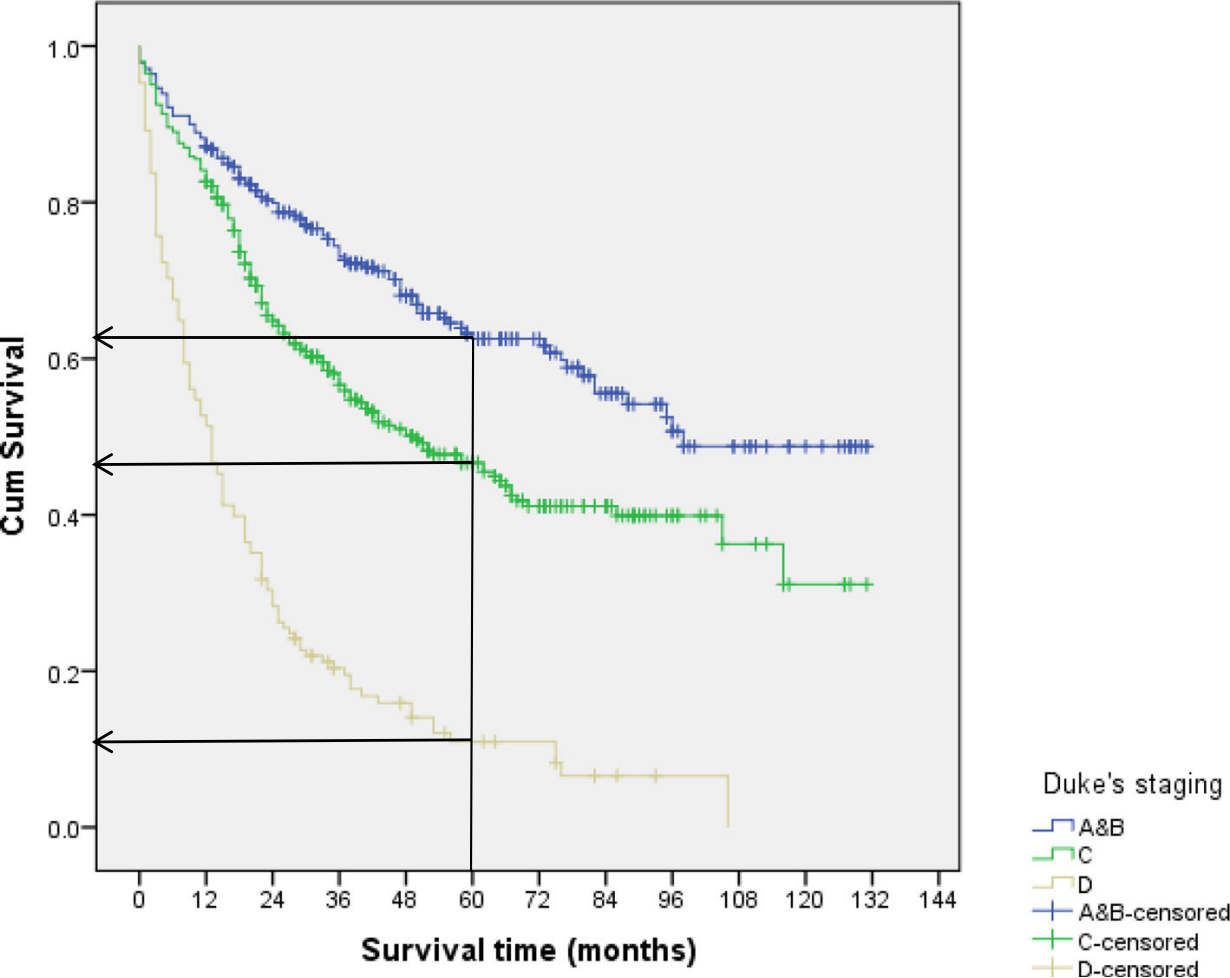
Survival curves of colorectal cancer by Duke’s staging

In the stratified analysis by ethnic groups (Table 3), patients from the Chinese ethnic group had significantly lower 5-year survival compared to the Indian and Malay patients in colon cancers (*p* = 0.039), and both colon and rectal cancers combined (*p* = 0.005). However, there was no significant difference in the 5-year survival rates for rectal cancer between ethnic groups. The 5-year survival rates for colorectal cancer patients with Duke’s stages A & B were higher (65.8%) compared to patients with advanced stages (Duke’s stages C, 49.4% and D, 12.6%) (*p* < 0.001) (Fig. 1). However, there was no significant difference in Duke’s stage by ethnic groups in the 5-year survival rates.

**Table 3:** Five-year survival of colorectal cancer patients by anatomic sites and Duke’s stage, stratified by ethnic groups

Clinical characteristics	Survival rate, % (95% CI)			
	^a^Malay (*n* = 225)	Chinese (*n* = 808)	Indians (*n* = 157)	p
Colorectal	48.50 (41.29–55.32)	39.68 (36.01–43.33)	47.49 (38.84–55.63)	0.039
Site:				
Colon	56.51 (44.75–66.69)	40.07 (34.86–45.22)	54.52 (42.18–65.29)	0.005
Rectum	43.87 (34.42–52.91)	39.81 (34.23–45.33)	36.91 (24.56–49.28)	0.674
Duke's stage^b^	Malay (*n* = 163)	Chinese (*n* = 546)	Indians (*n* = 105)	p
A & B	68.68 (51.43–80.88)	63.79 (55.88–70.66)	69.95 (51.73–82.39)	0.714
C	56.18 (42.43–67.86)	45.01 (37.96–51.80)	58.85 (41.56–72.61)	0.136
D	17.98 (6.27–34.53)	13.05 (7.03–20.96)	NA	-

*CI confidence interval, NA not available*

^a^ *3 ethnic groups ≠ 1212, as ethnic group, Others (n = 22) was excluded*

^b^ *Total sample with data on Duke’s stage, n = 814*

### Predictors of colorectal cancer survival

The total sample size used in the following analyses was 814 as cases with missing information on diagnosis of Duke’s staging (*n* = 398) were excluded. From the Cox proportional hazard regression model, predictors of 5-year survival for colorectal cancer patients were sex, ethnicity, pre-operative CEA levels, Duke’s stages, tumour grades and urgency of surgery (all *p* < 0.05); after adjusting for age, sex, comorbidities (diabetes mellitus and hypertension), tumour grades, treatment modalities and urgency of surgery (Table 4). The risk of colorectal cancer death was higher among male patients (HR: 1.41; 95% CI: 1.12, 1.76), Chinese ethnicity (HR:1.41; 95% CI: 1.07, 1.85), elevated pre-operative CEA levels (HR: 2.13; 95% CI: 1.60, 2.83), Duke’s stage C (HR: 1.68; 95% CI: 1.28, 2.21) and D (HR: 4.61; 95% CI: 3.39, 6.28); and patients with emergency surgery (HR: 1.52; 95% CI: 1.07, 2.15).

**Table 4:** Predictors of 5-year survival in colorectal cancer patients

Variables	^a^Crude HR (95% CI)	^b^Adjusted HR (95% CI)
Sex		
Female	1.00	1.00
Male	1.13 (0.96–1.31)	1.41 (1.12–1.76)
Ethnicity		
Malays	1.00	1.00
Chinese	1.26 (1.03–1.56)	1.41 (1.07–1.85)
Indians	1.06 (0.79–1.42)	1.32 (0.78–1.71)
Preoperative carcino-embryonic antigen (CEA) level		
Not elevated	1.00	1.00
Elevated	2.31 (1.91–2.79)	2.13 (1.60–2.83)
Duke’s stages		
A&B	1.00	1.00
C	1.66 (1.29–2.12)	1.68 (1.28–2.21)
D	4.94 (3.78–6.45)	4.61 (3.39–6.28)
Urgency of surgery		
Elective	1.00	1.00
Emergency	1.42 (1.11–1.82)	1.52 (1.07–2.15)

*HR* hazard ratio, *CI* confidence interval

^a^Univariate Cox Proportional Hazard Regression analysis

^b^Multivariate Cox Proportional Hazard Regression analysis, adjusted for age, gender, co-morbidities (diabetes mellitus, hypertension), tumour grades, and treatment modalities

Site-specific analyses for colon (*n* = 440) and rectal (*n* = 374) cancers after adjusting for confounders were also performed (Tables 5 and 6). Significant predictors for both colon and rectal cancer survivals were pre-operative CEA levels and Duke’s stages. The risks of colon and rectal cancer deaths for pre-operative CEA levels were 2.00 (95% CI: 1.30, 3.03) and 2.09 (95% CI: 1.39, 3.15) respectively. The hazard ratios for the diagnosis of tumour at Duke’s stage C and D were double in colon cancer (2.33 and 5.87) compared to rectal cancer (1.16 and 3.97). Ethnicity was only found to be significant in colon cancer survival, where Chinese (HR: 1.74; 95% CI: 1.07, 2.84) and Indian patients (HR: 1.88; 95% CI: 1.01, 3.51) had higher hazard ratios compared to Malays. On the other hand, males (HR: 1.68; 95% CI: 1.19, 2.37) and higher tumour grades (HR: 2.91; 95% CI: 1.65, 5.14) significantly predicted rectal cancer death.

**Table 5:** Predictors of 5-year survival in colon cancer patients

Predictors	^a^Crude HR (95% CI)	^b^Adjusted HR (95% CI)
Ethnicity		
Malays	1.00	1.00
Chinese	1.55 (1.10–2.17)	1.74 (1.07–2.84)
Indians	1.13 (0.71–1.78)	1.88 (1.01–3.51)
Preoperative CEA levels		
Not elevated	1.00	1.00
Elevated	2.58 (1.94–3.44)	2.00 (1.30–3.03)
Duke’s stages		
A&B	1.00	1.00
C	2.13 (1.49–3.05)	2.33 (1.56–3.49)
D	5.51 (3.73–8.16)	5.87 (3.73–9.24)

*HR* hazard ratio, *CI* confidence interval

^a^Univariate Cox Proportional Hazard Regression analysis

^b^Multivariate Cox Proportional Hazard Regression analysis, adjusted for age, gender, co- morbidities (diabetes mellitus, hypertension), tumour grades and treatment modalities

**Table 6:** Predictors of 5-year survival in rectal cancer patients

Predictors	^a^Crude HR (95% CI)	^b^Adjusted HR (95% CI)
Sex		
Female	1.00	1.00
Male	1.27 (1.02–1.60)	1.68 (1.19–2.37)
Preoperative carcino-embryonic antigen (CEA) level		
Not elevated	1.00	1.00
Elevated	2.03 (1.55–2.65)	2.09 (1.39–2.37)
Duke’s stages		
A&B	1.00	1.00
C	1.27 (0.89–1.80)	1.16 (0.79–1.72)
D	3.98 (2.37–5.80)	3.97 (2.59–6.10)
Tumour grades		
Low	1.00	1.00
High	2.99 (1.92–4.67)	2.91 (1.65–5.14)

*HR* hazard ratio, *CI* confidence interval

^a^Univariate Cox Proportional Hazard Regression analysis

^b^Multivariate Cox Proportional Hazard Regression analysis, adjusted for age, gender, co- morbidities (diabetes mellitus, hypertension), tumour grade and treatment modalities

## Discussion

The detailed characteristics of our colorectal cancer patients had been published elsewhere [12]. In brief, the demographic, clinical and treatment characteristics of our patients were comparable to other patients in Malaysia and some South East Asian countries [9, 11, 13, 14]. Our patients’ 1-, 3- and 5-year relative survival rates of colorectal cancer were also comparable to some countries in Asia [14–16]. Reports across Asia indicated that the 5-year survival rates ranged from 31.2% in India [17], 57.0 to 58.9% in Singapore [18], 41 to 61% in Korea [16] and 77% in China (highest) [19]. Although developed countries such as Australia, New Zealand, Canada, the United States and parts of Europe had the highest incidence rates of colorectal cancer, however their survival rates were better [20] than ours. Disparities in survival rates may be due to the different management practices and the effectiveness of implemented colorectal cancer screening programs among these countries.

On the other hand, there are not many local studies available for the comparison of colorectal cancer survival rates. Our 5-year colorectal cancer survival rate was slightly lower than the survival rate reported on patients recruited in 2008–2009 from the National Cancer Patient Registry-Colorectal Cancer data, which was 48.7% [21]. As a tertiary centre, patients referred to our hospital are usually of more advanced stage, as evidenced by nearly two-thirds of those with retrievable stage being Stage III or IV; this would skew the overall survival rates. When corrected for stage, a comparative study between hospitals from Kuala Lumpur and Kuching [10] showed that the 5-year colorectal cancer survival was lower for patients in Kuching compared to Kuala Lumpur. Another hospital from Kelantan, situated at the east coast of Peninsular Malaysia reported a lower 5-year survival rate for colorectal cancer (34.3%) [11] compared to our hospital. These discrepancies could be due to different stages at diagnosis or presentation as well as availability of expertise and facilities in different hospitals. Socio-economic status of patients from Kuala Lumpur or nearby location may be better than those from Kuching and Kelantan, as lower socio-economic status was a significant factor for late and more advanced stage at diagnosis, as well as poorer 3- and 5-year survival rates for colorectal cancers [10].

We did not find age, treatment modalities, diabetes mellitus and hypertension to be significant predictors of colorectal cancer survival. However, males had higher risks of death for colorectal cancer (Table 4). Contradicting results have been reported where some studies reported males to have poorer survival for colorectal cancer. [22, 23] while others did not find any difference in survival between sex [11, 21, 24, 25]. This may be related to the different proportions of colon and rectal cancer patients included in the different studies. In addition, different levels of health awareness and risk behaviours (smoking, physical inactivity etc.) as well as health seeking behaviours between genders [22] may be the contributing factors. Further research on these aspects should be conducted.

About two thirds of our patients were from the Chinese ethnic group. According to the findings from Abu Hassan et al. [4] on patients diagnosed from 2008 to 2013, those of Chinese ethnicity had the highest incidence for colorectal cancer (27.35 per 100,000), followed by the Malays (18.95 per 100,000), and Indians (17.55 per 100,000). The observed ethnic disparities may be due to the fact that our hospital is located in the Klang valley with more residents from the Chinese ethnic group compared to others. The ethnic distribution among patients in the hospital was also predominantly Chinese, thus giving rise to the observed results. The incidence of colorectal cancer among Chinese in our country was also higher than the Chinese from China [26]. This may be due to different lifestyle practices especially higher animal fat and red meat in their diet and physical inactivity. However, information on diet and physical activity levels were not available in our study.

Lowest survival rate was also observed among the Chinese compared to the Malay patients irrespective of anatomic site and Duke’s stages. These findings are in contrast with earlier reports in Malaysia and Singapore where non-Malays (including Chinese) were reported to have better survival rates [9, 11, 27]. Chinese were reported to have lower level of awareness in colorectal cancer compared to Malays [7] while another study reported that Malays had better recognition of symptoms [28] compared to the Chinese ethnic group. These could contribute to the late diagnosis or presentation at advance stage resulting poorer survival.

More advance Duke’s staging and elevated pre-operative CEA levels significantly predicted both colon and rectal cancers survival, similarly reported by other studies [11, 24, 29, 30]. However, given similar staging in comparison with patients from the developed countries, our patients’ survival was poorer [15, 27, 30], probably due to the different management practices and different patients’ compliance to treatment.

Emergency surgery was another predictor of poor colorectal cancer survival, as reported elsewhere [31]. Poor awareness on colorectal cancer [7, 28, 32] resulted in diagnosis at advanced stage with obstructive symptoms that needed emergency surgery. Effective health education campaigns need to be implemented to increase the awareness of our population. The programs should focus on increasing the knowledge on symptoms and risk factors of colorectal cancer, awareness on benefits of screening, and promotion of healthy life styles to prevent this disease. Primary care physicians should be given a bigger role in educating the public, as well as assist in identifying and recommending high-risk patients for early colorectal cancer screening and further expert management. Availability of programs for colorectal screening nationwide should also be provided.

Patients from the Malay ethnic group had better colon cancer survival than other ethnic groups as the Malay patients were younger [12] and hence had better survival. On the other hand, males had poorer survival for rectal cancer, as the narrow pelvis in males makes surgery more difficult technically and this may cause inadequate clearance in males compared to females. As expected, higher tumour grades significantly predicted rectal cancer survival.

### Limitations and strengths

Our study has a few limitations, which need to be considered while interpreting the results. As the data were extracted from the medical records, important information such as family history of colorectal cancer, socio-economic status, lifestyle practices such as smoking, diet and physical activity, adherence to treatment and follow-up/check-ups were unavailable. Future studies should be planned to collect these data. These data should also be routinely collected and recorded in the medical records. There were substantial missing data on clinical characteristics (such as Duke’s staging, pre-operative CEA levels, tumour grades etc.) in the medical records as these data were recorded in hard copies and had higher chance of being misplaced. However, documentation of these data should be improved with the implementation of electronic medical records. Future research addressing the above limitations should be conduted.

On the other hand, our study may have the largest number of patients from a single centre with a follow up duration of 10 years. Our study explored and provided multivariate analyses on a population substantially understudied and provided important ethnic comparisons.

## Conclusions

The survival rates of colorectal cancer among our patients were comparable with some Asian countries but lower than the developed countries. As expected, more advanced stage and late presentation were important predictors for colorectal cancer survival. A less expected finding was that males and patients from the Chinese ethnic group had lower survival rates compared to their counterparts. Therefore, health education programs targeting males and high risk ethnic groups, emphasizing the importance of screening and early diagnosis, recognition of symptoms and risk factors, should be implemented. Nationwide colorectal cancer screening programs should be made available, to increase early detection of colorectal cancer and improve the survival rates in the future.

## Abbreviations

CEA: Carcino-embryonic antigen
NRD: National Registration Department
NRIC: National Registration Identity Cards
UMMC: University Malaya Medical Centre
